# A Numerical Investigation on Hydrothermal Performance of Micro Channel Heat Sink with Periodic Spatial Modification on Sidewalls

**DOI:** 10.3390/mi13111986

**Published:** 2022-11-16

**Authors:** Nikita Kumari, Tabish Alam, Masood Ashraf Ali, Anil Singh Yadav, Naveen Kumar Gupta, Md Irfanul Haque Siddiqui, Dan Dobrotă, Ionela Magdalena Rotaru, Abhishek Sharma

**Affiliations:** 1 School of Automation, Banasthali Vidyapith, Jaipur 304022, India; 2 CSIR-Central Building Research Institute, Roorkee 247667, India; 3 Academy of Scientific and Innovative Research (AcSIR), Ghaziabad 201002, India; 4 Department of Industrial Engineering, College of Engineering, Prince Sattam Bin Abdulaziz University, Al-Kharj 16273, Saudi Arabia; 5 Mechanical Engineering Department, IES College of Technology, Bhopal 462044, India; 6 Mechanical Engineering Department, Institute of Engineering & Technology, GLA University, Mathura 281406, India; 7 Mechanical Engineering Department, King Saud University, Riyadh 11421, Saudi Arabia; 8 Faculty of Engineering, Department of Industrial Engineering and Management, Lucian Blaga University of Sibiu, 550024 Sibiu, Romania; 9 Department of Mechanical Engineering, Birsa Institute of Technology Sindri, Dhanbad 828123, India

**Keywords:** microchannel heat sink (MCHS), hydrothermal performance, thermal resistance, Nusselt number

## Abstract

Electronic gadgets have been designed to incorporating very small components such as microcontrollers, electronic chips, transistors, microprocessors, etc. These components are exceptionally heat sensitive and can be wrecked if heat is not released. As a result, the thermal control of such components is critical to their optimum performance and extended life. The use of a microchannel heat sink (MCHS) has shown promising solutions to remove the excess heat. In this paper, we have proposed a novel design of MCHS and investigated it numerically. Four different surface modifications on the sidewall of the passage, namely, extended triangular surface (ETS), extended circular surface (ECS), triangular groove surface (TGS), and the circular groove surface (CGS) in the passage of the microchannel have been exploited in the Reynolds number of 100–900. In the presence of geometrical modification, the cooling capacities have been enhanced. The results show that the Nusselt numbers of ETS-MCHS, ECS-MCHS, TGS-MCHS, and CGS-MCHS are increased by 4.30, 3.61, 1.62, and 1.41 times in comparison to the Nusselt number of MCHS with smooth passage, while the friction factor values are increased by 7.33, 6.03, 2.74, and 1.68 times, respectively. In addition, the thermohydraulic performance parameter (THPP) has been evaluated and discussed. The fact that MCHS have THPP values greater than unity demonstrates that the passage’s geometries are a practical means of achieving effective thermal management.

## 1. Introduction

In recent years, technological advancement plays a crucial role in our daily lives from home to workplaces everywhere. Electronic gadgets and microelectronics are the backbone of such technologies, which are applicable to the computing, automobile, telecommunication, mobile phone, robotics, and aerospace industries, as well as others [1,2]. The use of small electronic devices and microelectronics causes problems with heat dissipation. Therefore, thermal management is the key challenge to avoid the damage of such components. In this regard, microchannel heat sinks (MCHSs) are very popular and can dissipate the high densities’ heat flux and maintain the temperature below the threshold limit [3]. Its applicability is increased by the significant cooling properties and high fluid volume to heat transfer surface ratio in the MCHS. Proper cooling not only prevents damage to a heat sensitive component, it also improves its lifetime.

The main purpose of the MCHS is to increase natural and forced convection’s ability to transfer heat. Moreover, these heat transfer techniques have been employed in current heat exchangers [4,5,6,7]. These techniques can also be applied to improve the cooling capacities of the MCHS. The various approaches employed in MCHSs have been applied, such as geometrical modification in the passage of the microchannel [8,9,10], wavy channel [11,12], pin fins [13], porous media [14,15], nanofluids [3,16,17], extended surface [18], and fins [19].

The several experimental and numerical studies have been presented, wherein the roughness has been employed for better the mixing of the heat transfer fluid and to disturb the sublaminar layer, which leads to an improved convective heat transfer coefficient. In addition, the heat transfer area also increases in roughness [20]. Because of the pressure drop caused by the flow disturbance, there is a correspondingly higher need for pumping power [21]. Therefore, the optimum design of the microchannel is of utmost importance, particularly for a higher convective heat transfer coefficient and low pumping power [22]. The first study on an MCHS revealed that the hydraulic diameter has a significant effect on the heat transfer coefficient in comparison to the conventional heat exchanger [23]. Many other experimental and numerical investigations reported that the continuum theory for an incompressible flow in MCHS is able to predict the heat transfer mechanism [21,24].

Many experimental studies on MCHSs have been presented to establish the fact behind the heat transfer mechanism. Huang et al. [25] investigated an MCHS with and without fan-shaped re-entrant cavities in laminar flow. The results showed that an MCHS with re-entrant cavities is able to enhance the heat transfer rate and reduce the pressure drop. Moreover, a pressure-drop reduction effect has been found to increase the radius of re-entrant cavities. Qu and Mudawar [21] investigated a microchannel made of oxygen-free copper and fitted with a polycarbonate plastic cover plate which exploited deionized water as heat transfer fluid at two heat fluxes of 100 W/cm^2^ and 200 W/cm^2^. In an another experimental study, Chai et al. [26] investigated an MCHS with periodic expansion–constriction cross-sections. The results were analysed in terms of pressure drop, thermal resistance, and heat transfer due to periodic expansion–constriction cross-sections. Doan et al. [27] conducted the experiments to investigate heat dissipation and pressure drop behaviour considering the gravity and condensation of steam for both horizontal and vertical orientations of an MCHS. It was concluded that the performance index result of the vertical orientation was better compared to that of the horizontal orientation. Gaikwad and Mohite et al. [28] presented a study on the thermohydraulic characteristics of an MCHS equipped with cylindrical pins inserts. The effect of the pin diameter has been explored regarding thermohydraulic performance to determine the optimum pin diameter. The presence of pins completely alters the velocity distribution and increases heat transfer capacity while also increasing the pressure drop penalty.

To demonstrate the understanding of the fluid phenomenon that can correlate the heat transfer mechanism, numerous numerical studies on the performance of MCHS have been validated and presented. Kumar and Singh [29] investigated the fluid flow and heat transmission characteristics of single-phase flow in a microchannel with or without micro-inserts using computational fluid dynamic (CFD) analysis. The findings of this study revealed the fact that adding inserts and accelerating fluid flow strengthens heat transfer capabilities. Shui et al. [30] experimentally and numerically investigated the performance of an MCHS equipped with tree-like branching. The dimple was also exploited as turbulators for heat transfer improvement. The finding showed that the dimple was beneficial for increasing the average heat transfer rate of the branching microchannel. In addition, higher branching further increased heat transfer performance. Ambreen and Kim [31] numerically studied the performance of an MCHS due to the different nanoparticle sizes (20–200 nm) of Al_2_O_3_ and TiO that affected the thermos-physical properties of nanofluids. It was reported that the nanoparticle diameter had an inverse relationship with the convective heat transfer and the friction factor. Gunnasegaran et al. [32] investigated how the geometrical factors of the MCHS affected the flow and heat transmission characteristics. Rectangular, trapezoidal, and triangular cross-sections of the same corridor were used in three distinct ways. Additionally, it was determined that the heat sink with the smallest hydraulic diameter works better than the other heat sinks investigated in terms of pressure drop and friction factor. In another study, a dimple placed on the walls of an MCHS was tested experimentally for a Reynolds number ranging from 600 to 11000. The geometrical parameters considered were the ratio of the channel height to dimple print diameter and the ratio of the dimple depth to dimple print diameter. It was concluded that the local Nusselt number ratios also increased due to buoyancy and variable property influences [33].

Thermohydraulic performance is the key results of the microchannel heat sink, which combines the heat transfer enhancement and friction factor together. These parameters help to decide whether the design is viable or not. In this regard, Saha et al. [34] carried out a numerical simulation of the microchannel heat sink, wherein a right triangular groove on the side wall of the passage was considered as a geometrical modification. The effect of the angle of the right triangular grooves in the range of 15–75° has been studied according to its thermohydraulic performance parameter (THPP). The maximum THPP was found in the range of 3.40–4.16 at an angle of 15°. Vasta et al. [8] presented the numerical simulation of an MCHS which had a two-sided wedge at the base of the passage. It was reported that the wedge angle slightly influenced the Nusselt number; however, the friction factor did not show any deviation as a function of wedge angle. Moreover, the maximum THPP was found to be 1.045 when the wedge angle was 15°. Pandey and Singh [35] carried out numerical simulation and studied the effect of a Y-shaped insert with triangular perforation. The preformation index was considered to be in the range of 0–30%. The results indicate that the heat transfer is enhanced by 5.84 times. However, the maximum THPP was 3.25 at 30% for the performance index.

The thermophysical characteristics of the heat transfer fluid are also a factor in the mechanism of the enhanced heat transfer, in addition to the MCHS design. Many research studies have employed this specific fluid as a heat transfer fluid. These fluids are known as nanofluids, whose thermophysical properties can be altered. A review on nanofluid has been presented to discuss their suitability in the MCHS [36]. However, Al-Baghdadi et al. [3] highlighted that using nanofluids in a microchannel is unfeasible because water is more practical and less dangerous.

In the aforementioned discussion based on the literature survey, a passive technique in the form of geometrical modifications in an MCHS is a promising solution to remove the excess heat. Various geometrical modifications have been carried out to enhance the cooling capacity. The cross-section of the passage of the MCHS in several shapes (i.e., circular [37], two-sided wedge [8], rectangular [38], triangular [10], square [39]) have been investigated. Moreover, the passage of an MCHS with various elements such as pin [28], fillet ribs [22], oval-shaped pin fins [13], sinusoidal and wavy shape [40], dimple ribs [30,41], and an expansion–contraction passage [26] has been investigated with/without nanofluid. Further, there is scope to investigate the ribs of the different shapes in the passage of an MCHS. In this regard, four different periodic ribs in the MCHS passage, namely, extended triangular surface (ETS), extended circular surface (ECS), triangular groove surface (TGS), and circular groove surface (CGS), have been designed and simulated in the range of a Reynolds number of 100–900. Water is considered to be a heat transfer fluid which is best suited for an MCHS [3]. The objective of this work is to evaluate the heat transfer and friction characteristics and discuss the heat transfer mechanism. Moreover, thermohydraulic performance characteristics have been evaluated to find out the best configuration.

## 2. Details of MCHS Domain

### 2.1. Computational Domain

**Figure 1:**
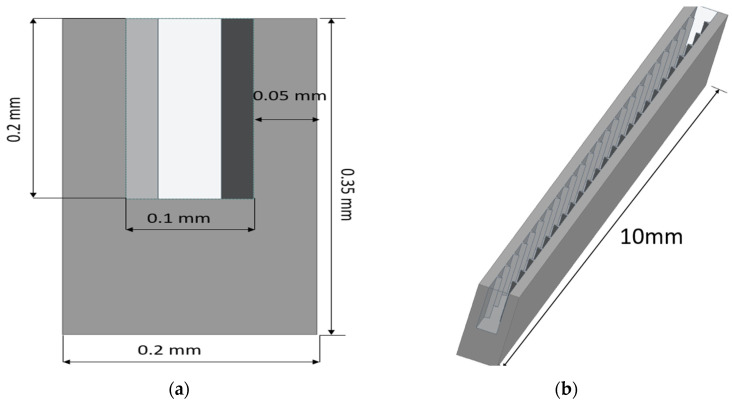

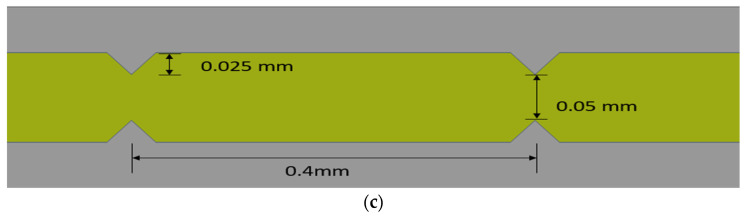
(**a**) Cross-sectional view, (**b**) isometric view, (**c**) top view of single passage of microchannel heat sink.

**Figure 2:**
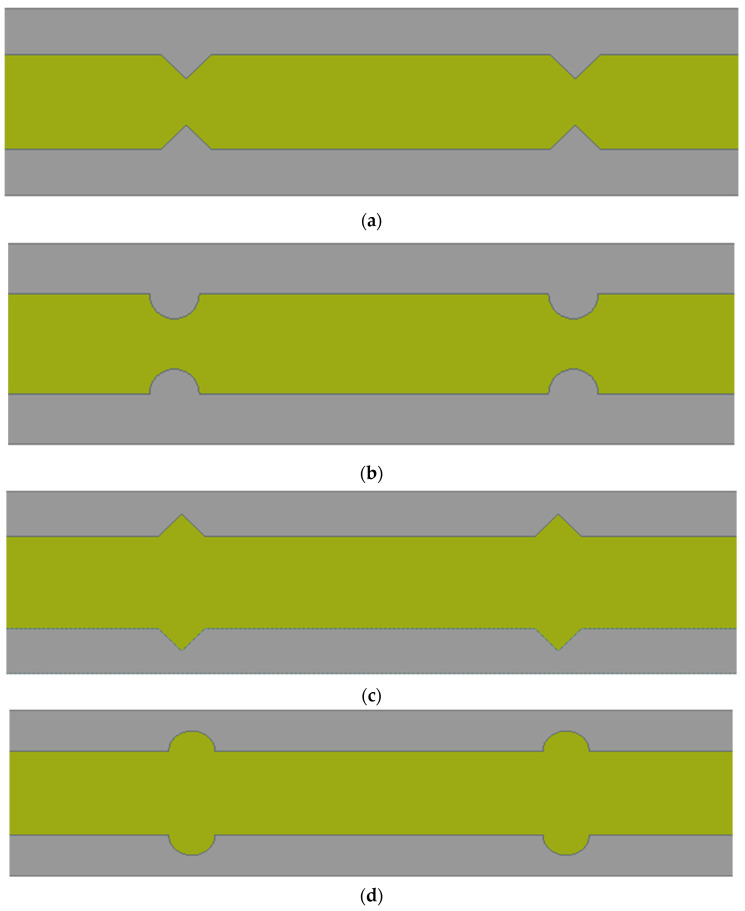
Top view of microchannel heat sink: (**a**) Type 1: extended triangular surface, (**b**) Type 2: extended circular surface, (**c**) Type 3: triangular groove surface, (**d**) Type 4: circular groove surface.

The computational domain of an MCHS along with heat transfer fluid are created using a design module of the Ansys workbench. A three-dimensional computational domain of the MCHS in a nonisothermal steady state has been considered in this study. The heat transfer fluid domain is exploited in a single phase. Since an MCHS is made up of 50 straight channels, enormous computational efforts and a long time are required for each simulation. In this regard, a single microchannel has been simulated to save time. The dimension of the single microchannel has been considered based on the experimental study [26]. The dimensions of length, width, and height are considered as 10 mm, 0.35 mm, and 0.2 mm, respectively. The pitch of the extended surface/groove has been employed as 0.4 mm, as suggested by Chai et al. [10]. As indicated by Chai et al. [10], one MCHS channel with the dimensions including a length of 10 mm, height of 0.35 mm, and of width 0.2 mm is therefore numerically simulated for all cases. According to research by Chai et al. [26], the pitch of the right triangular groove in the MCHS channel is estimated to be 0.4 mm. Since the route wall is 0.05 mm thick, the groove depth is calculated as 0.025 mm, or half of the thickness of the passage wall. Figure 1 shows the computational domain of the MCHS, wherein the dimensions of the length, width, height, and width of passage have been presented. The interface between the base of the MCHS and the fluid domain is planned, whereas the interface on the side walls of the passage and fluid domain follow the geometrical modification. Four different types of the sidewall modification, namely, Type 1—extended triangular surface (ETS), Type 2—extended circular surface (ECS), Type 3—triangular groove surface (TGS), and Type 4—circular groove surface (CGS), have been employed in this study. All four geometrical modifications on the sidewalls of flow passage have been shown in Figure 2.

### 2.2. Conservation Equations

Numerical simulation was carried out to exploit the following governing equations of continuity, momentum, and energy. The equations are solved for single-phase heat transfer fluid for laminar flow under steady state conditions. The water as a single-phase heat transfer fluid in incompressible form has been considered in this study. The following equations have been exploited in all simulations.

The conservation of mass or equation of continuity:

(1) $$\frac{\partial u}{\partial x}\;+\frac{\partial v}{\partial y}+\frac{\partial w}{\partial z}=0$$

where *u*, *v*, and *w* are velocity components in *x*, *y*, and *z* directions.

The conservation of momentum in *x* direction

(2) $$u\frac{\partial u}{\partial x}+v\frac{\partial u}{\partial y}+w\frac{\partial u}{\partial z}=-\frac{1}{\rho f}\frac{\partial p}{\partial x}+\frac{\mu f}{\rho f}\;(\frac{\partial^{2}u}{\partial x^{2}}+\frac{\partial^{2}u}{\partial y^{2}}+\frac{\partial^{2}u}{\partial z^{2}})$$

The conservation of momentum in *y* direction

(3) $$u\frac{\partial v}{\partial x}+v\frac{\partial v}{\partial y}+w\frac{\partial v}{\partial z}=-\frac{1}{\rho f}\frac{\partial p}{\partial y}+\frac{\mu f}{\rho f}\;(\frac{\partial^{2}v}{\partial x^{2}}+\frac{\partial^{2}v}{\partial y^{2}}+\frac{\partial^{2}v}{\partial z^{2}})$$

The conservation of momentum in *z* direction

(4) $$u\frac{\partial w}{\partial x}\;+v\frac{\partial w}{\partial y}\;+w\frac{\partial w}{\partial z}=-\frac{1}{\rho f}\frac{\partial p}{\partial z}+\frac{\mu_{f}}{\rho_{f}}\;(\frac{\partial^{2}w}{\partial x^{2}}+\frac{\partial^{2}w}{\partial y^{2}}+\frac{\partial^{2}w}{\partial z^{2}})$$

where $\rho_{f}$ is density (kg/m^3^) and $\mu_{f}$ is the dynamic viscosity of the heat transfer fluid. *p* is pressure (Pa).

The energy equation for heat transfer fluid

(5) $$u\frac{\partial T_{f}}{\partial x}\;+v\frac{\partial T_{f}}{\partial y}\;+w\frac{\partial T_{f}}{\partial z}=\frac{k_{f}}{\rho_{f}C_{P,f}}(\frac{\partial^{2}T_{f}}{\partial x^{2}}\;+\frac{\partial^{2}T_{f}}{\partial y^{2}}\;+\frac{\partial^{2}T_{f}}{\partial z^{2}})$$

where $C_{P,f}$ is specific heat capacity [J/kg⋅K] and $k_{f}$ is the thermal conductivity [W/m⋅K] of the heat transfer fluid. $T_{f}$ is temperature [K] of the heat transfer fluid.

The energy equation for MCHS (silicon)

(6) $$k_{mc}\left(\frac{\partial^{2}T_{mc}}{\partial x^{2}}+\frac{\partial^{2}T_{s}}{\partial y^{2}}+\frac{\partial^{2}T_{s}}{\partial z^{2}}\right)=0$$

where *k_mc_* is for thermal conductivity of the silicon material [W/m⋅K]. *T_mc_* is the temperature of the MCHS.

### 2.3. Thermophysical Properties of MCHS and Fluid Domain

Two computational domains, i.e., an MCHS and fluid domains, have been incorporated in the simulations. The silicon is the best suited material in the fabrication of the MCHS [42]; therefore, silicon material has been provided in the MCHS domain. Since heat transfer fluid is considered as water, the thermophysical properties of water have been employed in the simulation. Table 1 and Table 2 show the thermophysical properties of the heat transfer fluid and MCHS (silicon), respectively.

### 2.4. Boundary Conditions and Computational Procedure

For velocity–pressure coupling in the multi-grid solution based on the finite volume method using Ansys Fluent, the Semi-Implicit Method for Pressure Linked Equations (SIMPLE) algorithm was used. The iterative procedure, with a convergence criteria of 1.0 × 10^−9^, 1.0 × 10^−6^, and 1.0 × 10^−6^, has been applied in energy, velocity component, and momentum, respectively. In order to begin the iterative method, initial and boundary conditions are specified. Inlet velocity conditions are provided to the fluid at the inlet, which is specified using a Reynolds number in the range of 100–900 at a temperature of 300 °K. Zero-gauge pressure is provided to the fluid at the outlet of the MCHS, which represents the atmospheric condition. A uniform heat flux of 1 × 10^6^ W/m^2^ is applied on the bottom wall of the MCHS. Boundary conditions of the side walls of the MCHS is considered as symmetrical. The boundary condition also listed in Table 3.

### 2.5. Meshing and Grid Independent Test

**Figure 3:**
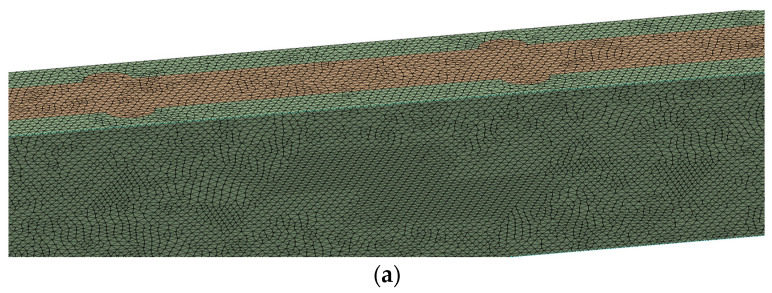

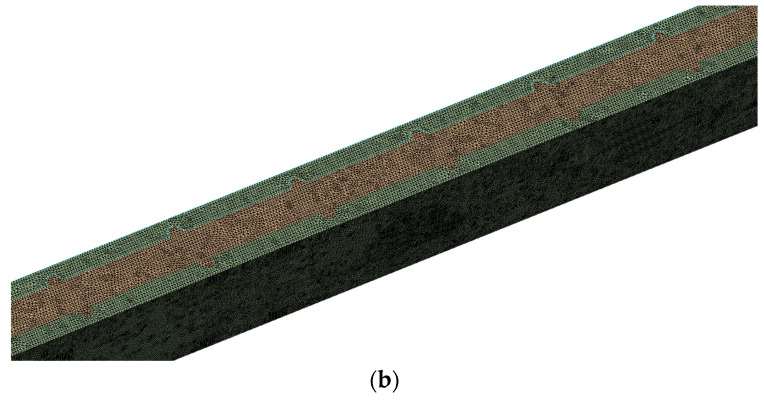
Meshing of fluid and MCHS domain: (**a**) enlarged view, (**b**) isometric view.

A three-dimensional domain of a single MCHS with fluid was created in the design module of the Ansys workbench. Domains are discretized to create the unstructured tetrahedron mesh. The meshing of the MCHS and fluid has been shown in Figure 3. Meshing is very fine to resolve boundary layer near the surface zone. Many simulations have been carried out to show the mesh sensitivity on solutions. Several simulations were conducted by varying the number of nodes and elements from 628,778 to 1,989,479 and 3,251,880 to 10,808,376, respectively. In order to show the grid independency of the solution, Table 4 shows the effect of the number of nodes and elements on the friction factor. It can be seen that percentage error in the friction factor decreased from 0.25% to 0.098% when the number of nodes and elements increased. This indicates that a further increase in the number of nodes and elements does not affect the friction factor, and therefore that the number of nodes and elements does not have a considerable effect on the friction factor. Based on this grid independent test, 1,989,479 nodes have been selected for further simulations.

### 2.6. Data Reduction and Formulation

Utilizing the numerical solution in the flow and microchannel domain, the various data are gathered. In the MCHS, data are transformed into meaningful findings in terms of the Nusselt number, friction factor, and flow pumping power. To demonstrate the performance, the following parameters were analysed:

Reynolds number

(7) $$\;Re=\frac{\rho_{f}{µ}_{m}D_{h}}{\;{µ}_{f}}\;\;$$

The hydraulic diameter

(8) $$D_{h}=\frac{2\;W\;H}{W+H}\;\;$$

The average friction factor

(9) $$\bar{f\;}=\frac{\Delta p\;D_{h}}{2\;\rho_{f}\;L\;u_{m}^{2}}$$

Average heat transfer coefficient

(10) $$\bar{h\;}=\frac{q\;A_{q}}{A_{c}\left(\bar{T_{c}}-\bar{T_{f}}\right)}8$$

Conjugated area average temp

(11) $$\;\bar{T_{c}}=\frac{\int T\;dA}{\int dA\;}\;$$

Avg temp of heat transfer fluid

(12) $$\;{\bar{T\;}}_{f}=\frac{\int T\;\rho_{f}\;dV}{\int \;\rho_{f}\;dV}$$

The average Nusselt number

(13) $$\bar{Nu}=\frac{\bar{\;h}\;D_{h}}{k_{f}\;\;}\;\;$$

Friction factor

(14) $$\bar{f}=\frac{\Delta p\cdot \;D_{h}}{2\cdot \rho_{f}\cdot L\cdot v^{2}\;}$$

### 2.7. Model Validation

**Figure 4:**
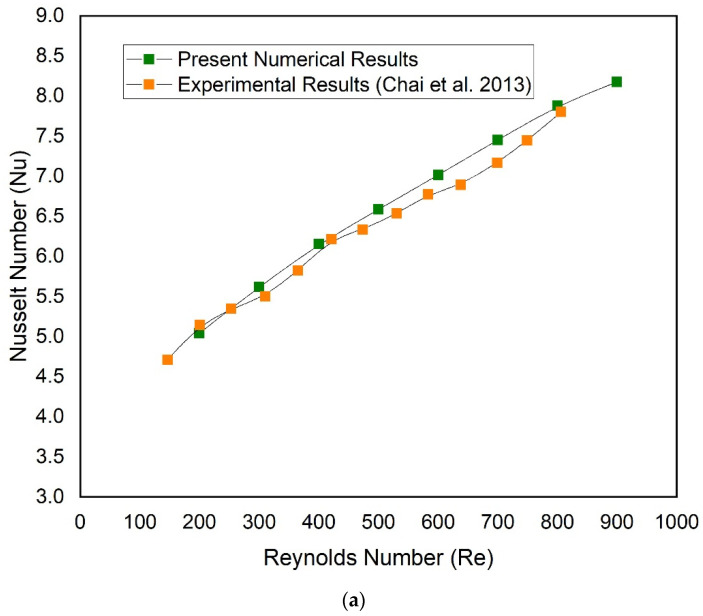

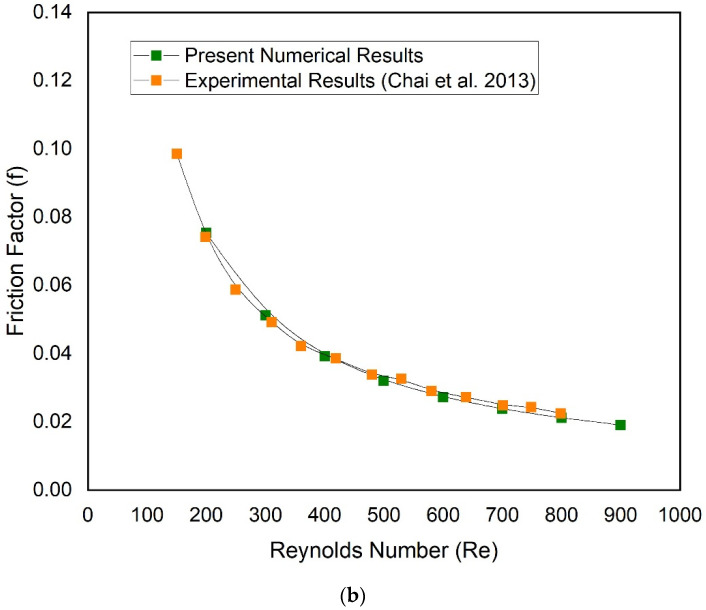
Comparison of numerical results with corresponding experimental [26]: (**a**) Nusselt number, (**b**) friction factor.

**Figure 5:**
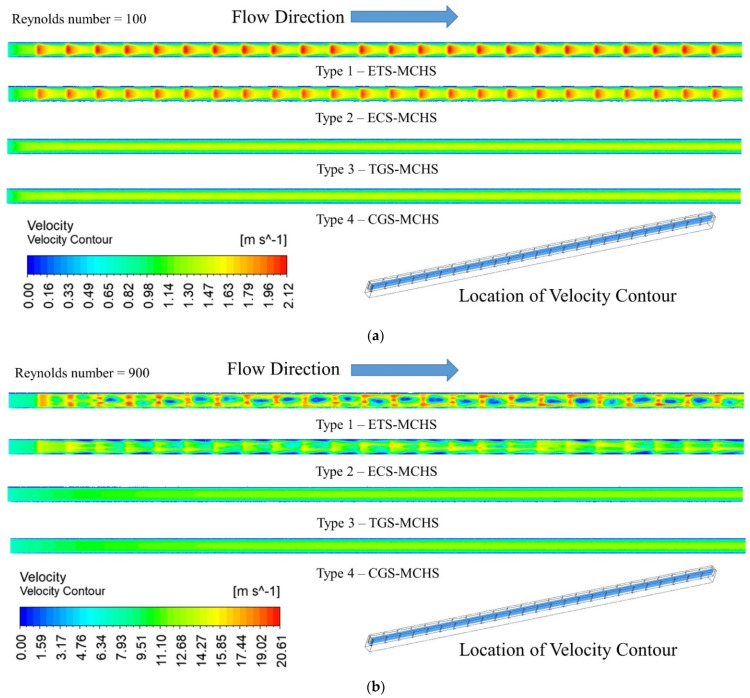
Velocity contour of heat transfer fluid at mid plane of fluid domain for (**a**) *Re* = 100 and (**b**) *Re* = 900.

Simulation of a single microchannel (traditional microchannel) without any geometrical modification is carried out to validate the present numerical results. The present numerical results of the Nusselt number and friction factor have been compared with a well-known experimental study conducted by Chai et al. [26]. Figure 4 and Figure 5 include a comparison of the Nusselt number and friction factor, respectively. It is evident that the current numerical results and the corresponding experimental data are in good agreement, demonstrating the validity of the results for further simulations. 

## 3. Result and Discussion

The CFD simulation of an MCHS having four types of different geometrical modifications, namely, extended triangular surface (ETS), extended circular surface (ECS), triangular groove surface (TGS), and circular groove surface (CGS) in the flow passage of an MCHS are performed according to a Reynolds number in the range of 100–900. The corresponding velocity of the fluid is provided to achieve the respective Reynolds number. The constant uniform heat flux of 1 × 10^6^ W/m^2^ is supplied to the base of the MCHS. In order to analyse the results, contours of the velocity, pressure, and temperature have been presented. The numerical data of the heat transfer and friction characteristics have been presented in term of Nusselt number, friction factor, and pressure drop as follows.

### 3.1. Velocity Distribution

For Reynolds numbers of 100 and 900, the velocity contours of the fluid flow domain for each of the four types of MCHS have been presented at the mid plane of the domain in Figure 5. In order to show the comparison, the legend of the velocity range remained same for all MCHS. It can be clearly seen that the velocity passed to the triangular extended surface is highest periodically in the case of Type 1 (ETS-MCHS) and Type 2 (ECS-MCHS) for both Raynolds number (100 and 900). The maximum velocities of 2.12 and 20.61 are observed in the case of Type 1 (ETS-MCHS) at a Reynolds number of 100 and 900, respectively. The maximum velocities in the case of Type 2 (ECS-MCHS) slightly decreased to 2.04 and 17.46 at a Reynolds number of 100 and 900, respectively. In addition to this, periodic velocity for Type 3 (TGS-MCHS) and Type 4 (CGS-MCHS) are not observed, which indicates that Type 3 (TGS-MCHS) and Type 4 (CGS-MCHS) do not alter the flow significantly. The maximum velocity in the case of Type 3 (TGS-MCHS) and Type 4 (CGS-MCHS) are similar, i.e., 1.48 at a Reynolds number of 100. However, the maximum velocity in the case of Type 3 (TGS-MCHS) and Type 4 (CGS-MCHS) are slightly different, i.e., 12.68 and 12.66, respectively.

### 3.2. Pressure Drop Distribution

**Figure 6:**
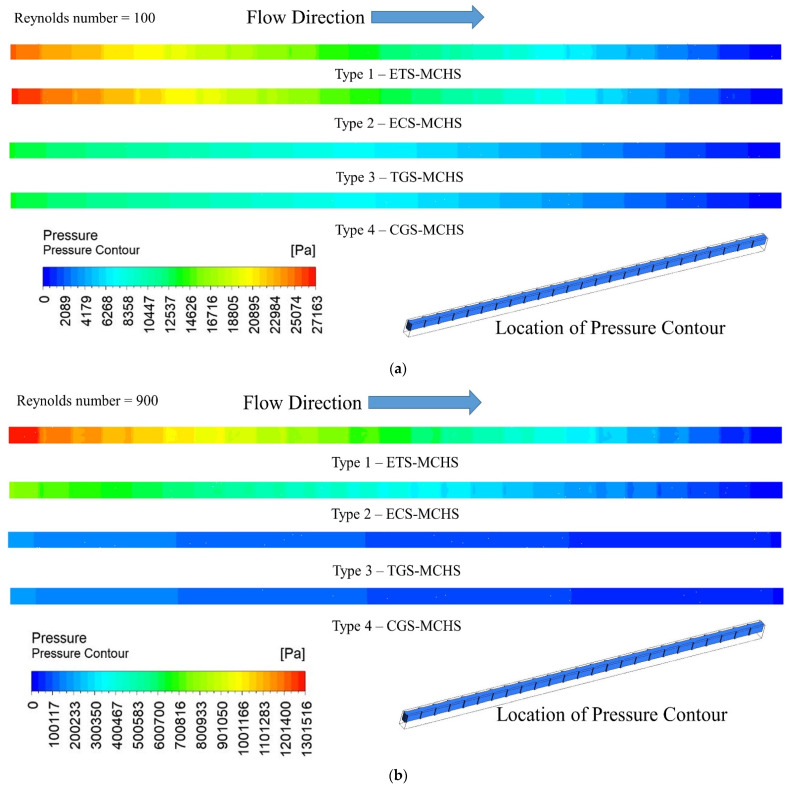
Pressure contour of heat transfer fluid in fluid domain for (**a**) *Re* = 100 and (**b**) *Re* = 900.

The pressure contours in the flow domain of the fluid of all four types of MCHS have been presented for a Reynolds number of 100 and 900 in Figure 6. The contours clearly show that a pressure drop of fluid in the passage of all MCHSs decreased continuously along the flow for both Reynolds numbers of 100 and 900. Moreover, it can be seen that the maximum pressure drop is observed in the case of Type 1 (ETS-MCHS) for both Reynolds numbers. In the case of the Type 2 (ECS-MCHS) pressure drop, it slightly decreased in comparison to the pressure drop in Type 1 (ETS-MCHS). Similarly, very small pressure drops are observed in the case of Type 3 (TGS-MCHS) and Type 4 (CGS-MCHS) for both a Reynolds number of 100 and 900. Moreover, there are significant differences in the pressure drops observed for all types of MCHSs when compared at a Reynolds number of 100 and 900. Large pressure drops in the flow indicate that higher pumping power is required to achieve the same flow. Therefore, Type 1 (ETS-MCHS) and Type 2 (ECS-MCHS) required comparatively large pumping power compared to Type 3 (TGS-MCHS) and Type 4 (CGS-MCHS). 

### 3.3. Temperature Distribution

**Figure 7:**
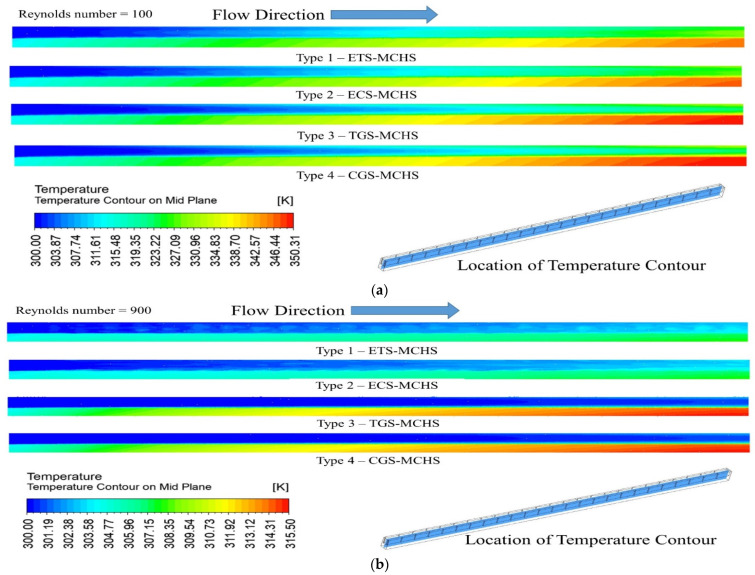
Temperature contour of heat transfer fluid and MCHS (at mid plane) for (**a**) *Re* = 100 and (**b**) *Re* = 900.

The temperature contour on the mid plane of the fluid and microchannel domains has been presented for a Reynolds number of 100 and 900, as shown in Figure 7. The bottom domain of the temperature shows the domain of the microchannel, while the upper domain shows the domain of the fluid. It has been clearly shown that the highest temperature is observed in the microchannel domain as compared to the fluid domain. Moreover, the temperature of the domain increased from the fluid inlet to the fluid outlet in all types of MCHSs at both Reynolds numbers, as expected. A maximum temperature of 350.31 °K was found in the case of Type 4 (CGS-MCHS) and a minimum temperature of 345.19 °K was found in the case of Type 1 (ETS-MCHS) at a Reynolds number of 100. Similarly, a maximum temperature of 315.50 °K was found in the case of Type 4 (CGS-MCHS) and a minimum temperature of 311.94 °K was found in the case of Type 1 (ETS-MCHS) at a Reynolds number of 900. This significant variation in temperature in the case of Type 1 (ETS-MCHS) and Type 4 (CGS-MCHS) at both Reynolds numbers is considerable and indicates that the extended triangular surface disturbed the flow and is responsible for the higher heat dissipation rate. Because of the noticeably higher temperature, it is clear that the rate of heat dissipation is low [8]. In addition to this, there is a significant temperature difference in all types of MCHSs when the Reynolds number changed from 100 to 900. This is due to fact the that higher Reynolds numbers supress the sublaminar layer, which can lead to a higher heat dissipation rate.

**Figure 8:**
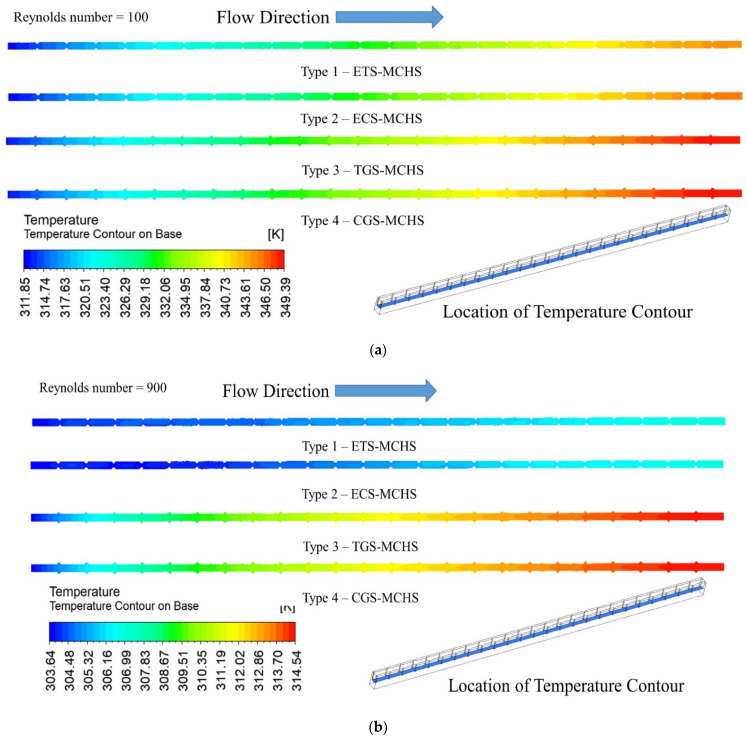
Temperature contour of MCHS (at base of passage) for (**a**) *Re* = 100 and (**b**) *Re* = 900.

The temperature contour on the base of the MCHS has been presented for all four types of MCHS at a Reynolds number of 100 and 900 in Figure 8. The location of the contours has also been in shown for a better understanding. It can be seen from these contours that the temperature increases for all types of MCHSs at both Reynolds numbers. The following maximum temperature in the case of Type 1 (ETS-MCHS), Type 2 (ECS-MCHS), Type 3 (TGS-MCHS), and Type 4 (CGS-MCHS) are 345.19 °K, 346.25 °K, 349.13 °K, and 349.39 °K, respectively, at a Reynolds number of 100. Similarly, the maximum temperatures of the respective MCHSs are 307.06 °K, 307.11 °K, 314.52 °K, and 314.54 °K at a Reynolds number of 900. The lowest temperature in the case of Type 1 (ETS-MCHS) for both Reynolds numbers indicates that the cooling capacity of this particular MCHS is highest among all MCHSs. In addition to this, the temperatures of the respective MCHSs significantly decrease when the Reynolds number changed from 100 to 900. This notable drop in maximum temperature shows the role of the Reynolds number, which has a significant effect on the dissipated rate from the MCHS.

### 3.4. Performance Analysis

In the aforementioned subsection, velocity, pressure, and temperature contours have been presented for all types of MCHSs at a Reynolds number of 100 and 900. The respective contours have been compared to show the effect of geometrical modifications. In order to establish the fact due to geometrical modification, numerical results in terms of pumping power, friction factor, Nusselt number and thermohydraulic performance parameters have been presented and discussed in the following subsections. 

**Figure 9:**
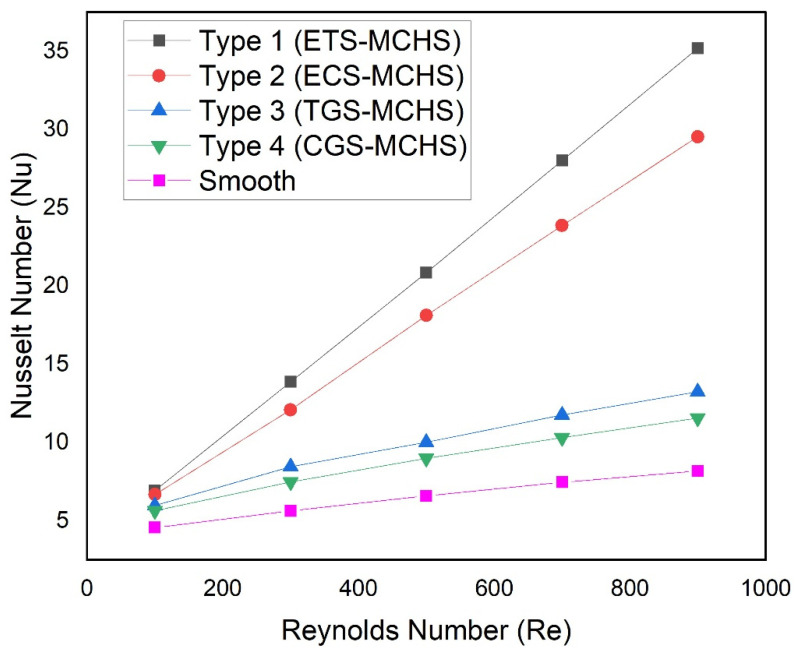
Plots of Nusselt number with Reynolds numbers for different design types.

Figure 9 shows the relationship between the Nusselt number and the Reynolds number for all types of MCHSs. The trend of the Nusselt number with Reynolds number steadily increases for all types of MCHSs. This trend of the Nusselt number is expected. The Nusselt number of Type 1 (ETS-MCHS) and Type 2 (ECS-MCHS) linearly increase with the Reynolds number. However, the Nusselt number of Type 3 (TGS-MCH) and Type 4 (CGS-MCHS) do not increase linearly with the Reynolds number. The slope of the Nusselt number decreases slightly with an increase in Reynolds number. This may be due to fact that the extended surface in Type 1 (ETS-MCHS) and Type 2 (ECS-MCHS) contribute to more turbulence in comparison to Type 3 (TGS-MCH) and Type 4 (CGS-MCHS). Therefore, the Nusselt number is found to be linearly increased in the case of the extended surface. Moreover, the increment rate of the Nusselt number is highest in the case of Type 1 (ETS-MCHS), followed by Type 2 (ECS-MCHS) and Type 3 (TGS_MCH), while Type 4 (CGS-MCHS) has the lowest increment rate of the Nusselt number. The Nusselt numbers are varied in the ranges of 6.92–35.19, 6.67–29.53, 5.93–13.24, and 5.63–11.55 in the case of Type 1 (ETS-MCHS), Type 2 (ECS-MCHS), Type 3 (TGS-MCH), and Type 4 (CGS-MCHS), respectively. There is significant variation in the Nusselt numbers due to the extended surface and groove, which can be explained by the temperature in MCHSs.

**Figure 10:**
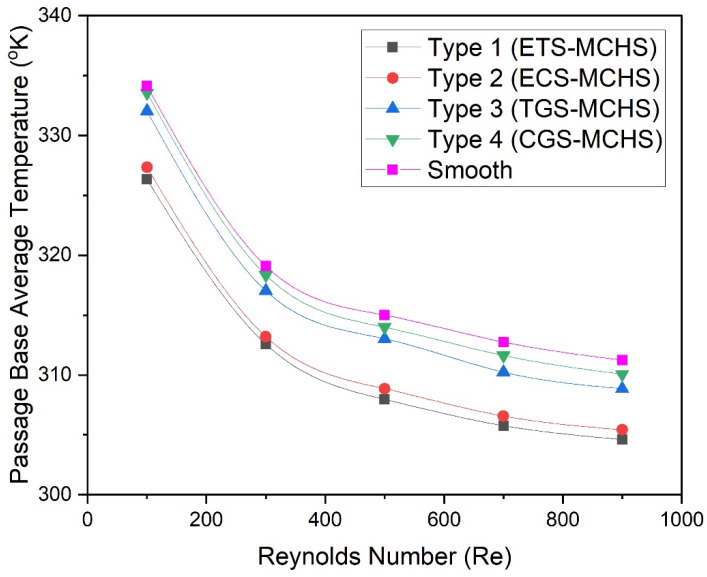
Plots of average temperature at base of passage of MCHS with Reynolds numbers for different design types.

In this regard, Figure 10 shows the variation of the average temperature of the base of the passage of the MCHS as a function of the Reynolds number. It can be seen that the temperature of the base of all types of MCHSs decrease with in Reynolds number. In the beginning, the temperature decreases rapidly; thereafter, the temperatures decrease steadily for all types of MCHSs. However, the temperature of the base of Type 4 (CGS-MCHS) has the highest values, followed by the temperature of the base of Type 3 (TGS-MCH) and Type 2 (ECS-MCHS). Type 1 (ETS-MCHS) has the lowest values among all the MCHS types. The lowest values of the temperature in the case of Type 1 (ETS-MCHS) indicate a higher heat dissipation rate, leading to the highest Nusselt number.

**Figure 11:**
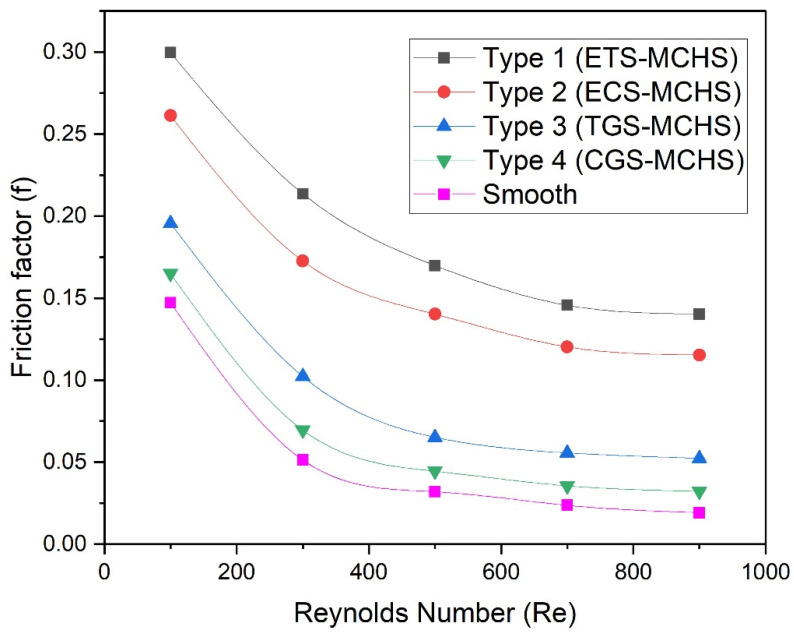
Plots of friction factor with Reynolds numbers for different angles.

In the previous subsection, pressure contour due to all types of MCHSs have been presented and discussed. In order to establish the fact concerning the pressure drop, the variations of the friction factor for all types of MCHSs have been presented as a function of the Reynolds number as shown in Figure 11. It can be seen that the pressure drop in all types of MCHSs decreases with an increase in the Reynolds number. In the beginning, the friction factor decreases rapidly; thereafter, it becomes constant. In addition to this, the friction factor is found to be maximum in the case of Type 1 (ETS-MCHS), followed by Type 2 (ECS-MCHS), and Type 3 (TGS_MCH), while Type 4 (CGS-MCHS) has the lowest friction factor. Friction factors are varied in the ranges of 0.300–0.140, 0.216–0.115, 0.196–0.0520, and 0.165–0.032 in the case of Type 1 (ETS-MCHS), Type 2 (ECS-MCHS), Type 3 (TGS-MCH), and Type 4 (CGS-MCHS), respectively.

**Figure 12:**
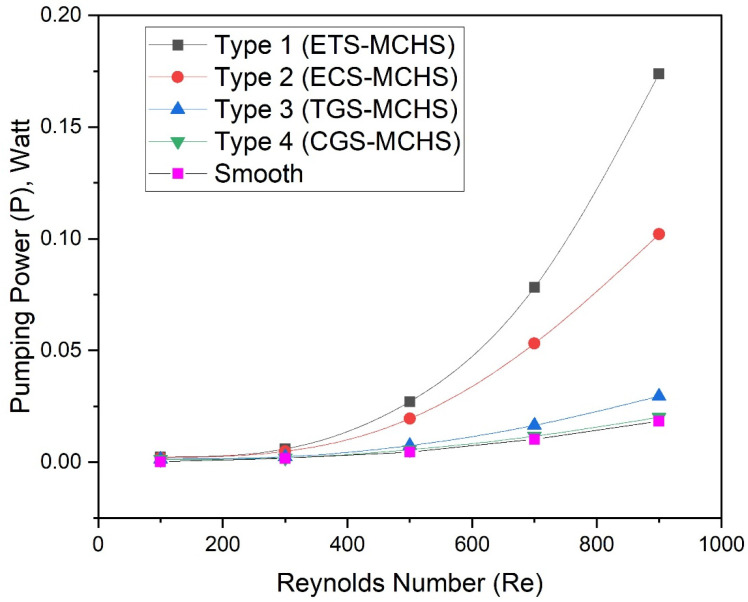
Plots of pumping power with Reynolds numbers for different design types.

The highest friction factor in the case of Type 1 (ETS-MCHS) is found due to the extended surface, which disturbed the flow due to the sharp edge. A higher fiction factor requires more pumping power. In order to ensure this, the pumping power of all types of the ribs has been evaluated and presented as a function of the Reynolds number, as shown in Figure 12. It can be clearly seen that the pumping power of all types of MCHSs increases with an increase in the Reynolds number. However, the large variance in pumping power can be seen as an effect of geometrical modification. In the case of Type 1 (ETS-MCHS), the pumping power increases exponentially, while the pumping power of Type 3 (TGS_MCH) and Type 4 (CGS-MCHS) do not increase significantly.

In the aforementioned discussion, both the Nusselt number and friction factor have been found to be higher in comparison to Type 2 (ECS-MCHS), Type 3 (TGS-MCH), and Type 4 (CGS-MCHS). It is very difficult to decide which is the best configuration based on the Nusselt number and friction factor. In order to analyse the best configuration, thermal and hydraulic performance, which show the cumulative performance, need to be considered together. In this regard, the thermohydraulic performance parameter (THPP), η, has been introduced to show the overall performance due to different geometrical modifications in the flow passage of the MCHS. If THPP values are more than one, the total outcome will almost certainly be productive, irrespective of the particular Nusselt number and friction factor values. THPP is given below:

(15) $$\eta =(Nu/Nus)/{\left(f/f_{s}\right)}^{1/3}$$

**Figure 13:**
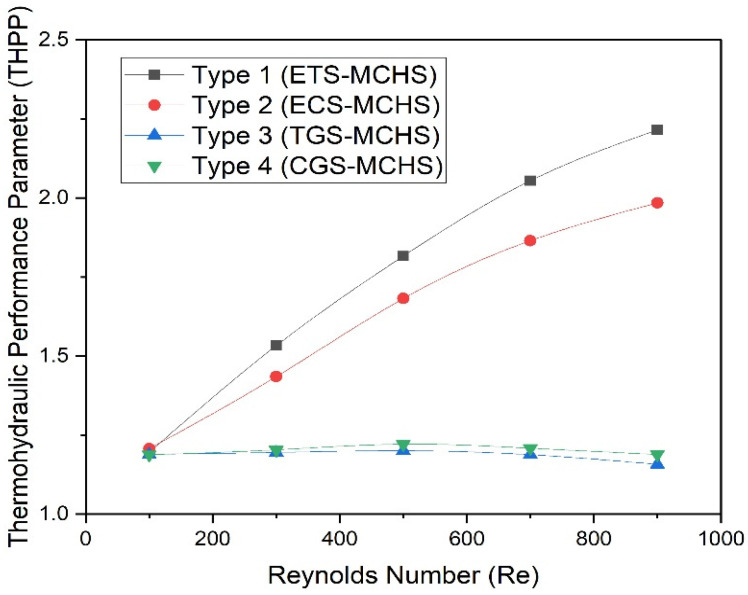
Plots of thermohydraulic performance parameter (*η*) with Reynolds numbers for different design types.

THPP values for different types of MCHS have been plotted as a function of the Reynolds number, as shown in Figure 13. It can be seen that THPP values increase with an increase in the Reynolds number in the case of Type 1 (ETS-MCHS) and Type 2 (ECS-MCHS); THPP values do not change with the Reynolds number in the case of Type 3 (TGS-MCH) and Type 4 (CGS-MCHS). The values of the THPP varies in the range of 1.20–2.21, 1.21–1.98, 1.16–1.20, and 1.19–1.22 in the case of Type 1 (ETS-MCHS), Type 2 (ECS-MCHS), Type 3 (TGS-MCH), and Type 4 (CGS-MCHS), respectively. In addition to this, THPP values are greater than one for all types of MCHSs in the selected range of the Reynolds number. This indicates that the design of the MCHSs have advantages over the MCHS without geometrical modification. The design of Type 1 (ETS-MCHS) is found to be the best configuration among all of the configurations investigated in this study.

## 4. Conclusions

The heat transfer mechanism and friction factor characteristics of laminar flow in the passage of an MCHS having geometrical modifications in the form of an extended triangular surface (ETS), extended circular surface (ECS), triangular groove surface (TGS), and circular groove surface (CGS) have been study numerically. The results of all four geometries have been compared and discussed according to Reynolds numbers ranging from 100 to 900. Some of the important conclusions are summarized below:

- The highest Nusselt number has been observed in the case of the Type 1 (ETS-MCHS) in the range of 6.92–35.19, while lowest Nusselt number was observed in the case of Type 2 (ECS-MCHS) in the range of 5.63–11.55. Similarly, the highest friction factor has been observed in the case of Type 1 (ETS-MCHS) in the range of 0.3–0.140, while lowest was observed in the case of Type 2 (ECS-MCHS) in the range of 0.165–0.032;
- Nu and f enhancement factors have been determined. The maximum enhancement of Nu and f are found to be 4.3 and 7.33 in the case of Type 1 (ETS-MCHS), while the minimum enhancement of Nu and f are found to be 1.23 and 1.12 in the case of Type 4 (CGS-MCHS);
- Pumping power is showed to be significantly higher for Type 1 (ETS-MCHS) and Type 2 (ECS-MCHS), whereas Type 3 (TGS-MCH) and Type 4 (CGS-MCHS) geometries do not contribute to considerable pumping power;
- Comparatively higher thermal performance parameters (THPPs) are found for Type 1 (ETS-MCHS) and Type 2 (ECS-MCHS), which have advantages over Type 3 (TGS-MCH), Type 4 (CGS-MCHS), and those without geometrical modifications.

## Figures and Tables

**Table 1:** Heat transfer fluid (water): the thermophysical properties.

Fluid	Density [kg/m^3^]	Dynamic Viscosity μ [Pa·s]	Specific Heat Cp [J/kg·K]	Thermal Conductivity k [W/m·K]
Water	998.2	0.001	4182	0.60

**Table 2:** Microchannel heat sinks (MCHS) (silicon): the thermophysical properties.

Material	Density ρs [kg/m^3^]	Poisson’s Ratio α	Specific Heat Cps [J/kg·K]	Thermal Conductivity ks [W/m·K]	Thermal Expansion β [1/K]	Young’s Modulus Es [Pa]
Silicon	2329	0.28	700	130	2.6 × 10^−6^	170 × 10^9^

**Table 3:** Boundary Conditions.

Location	Boundary Conditions
Inlet	Velocity inlet based on the Reynolds number at 300 °K
Outlet	Zero pressure gauge
Bottom of the MCHS	Heat flux 1.0 × 10^6^ W/m^2^
Side Walls of MCHS	Symmetry

**Table 4:** Grid independent test.

Serial Number	Nodes	Element	Friction Factor (f)	Percentage Variation in Friction Factor (f)
1	6,28,778	32,51,880	0.1569	-
2	10,59,696	55,99,292	0.1573	0.25
3	14,30,704	76,57,556	0.1575	0.135
4	19,89,479	1,08,08,376	0.1577	0.098

## Data Availability

Data available on request.

## Nomenclature

A_c_	Convective heat transfer area (mm^2^)
A_q_	Base Area of Channel (mm^2^)
C_p_	Specific Heat (J/kg⋅K)
D_h_	Hydraulic Diameter (mm)
E	Young Moduls (GPa)
f	Friction Factor
L	Length (mm)
h	Convective eat transfer coefficient (W/mm^2^·°K)
H	Height of passage (mm)
k	Thermal Conductivity (W/m·°K)
Nu	Nusselt Number
p	Pressure (Pa)
∆p	Pressure Drop
P	Pumping power (w)
q	Heat Flux (W/m^2^)
Re	Reynolds Number
T	Temperature (K)
u	Velocity (m/s)
W	Width of microchannel (m)
**Greek letters**	
α	Poisson’s ratio
β	Thermal expansion [1/K]
μ	Dynamic Viscosity (N·s/m^2^)
η	Thermohydraulic performance parameters (THPP)
ρ	Density (kg/m^3^)
**Subscripts**	
in	Inlet
o	Outlet
mc	Microchannel base
f	Fluid (coolant)
s	Conventional
